# Clinical characteristics of *Plasmodium vivax* malaria infection in children and adolescents in the Republic of Korea during the period 2000 to 2016: a retrospective study

**DOI:** 10.1186/s12879-025-10501-9

**Published:** 2025-01-21

**Authors:** Jung Ah Lee, Je Eun Song, Seong Yeon Park, Yoon Soo Park, Yoonseon Park, Yee Gyung Kwak, Sang-Eun Lee, Hyun-Il Shin, Joon-Sup Yeom

**Affiliations:** 1 Division of Infectious Diseases, Department of Internal Medicine, Yonsei University College of Medicine, 50-1, Yonsei-Ro, Seodaemun-Gu, Seoul, 03722 Republic of Korea; 2 Division of Infectious Diseases, Inje University Ilsan Paik Hospital, 170, Juhwa-ro, Ilsanseo-gu, Goyang-si, 10380 Gyeonggi-do Republic of Korea; 3 Division of Infectious Diseases, Dongguk University Ilsan Hospital, 27, Dongguk-ro, Ilsandong-gu, Goyang-si, 10326 Gyeonggi-do Republic of Korea; 4 Division of Infectious Disease, Department of Internal Medicine, Yongin Severance Hospital, Yonsei University College of Medicine, 363, Dongbaekjukjeon-daero, Giheung-gu, Yongin-si, Gyeonggi-do 16995 Republic of Korea; 5 Division of Infectious Diseases, Department of Internal Medicine, Gil Medical Center, Gachon University College of Medicine, 783, Namdong-Daero, Namdong-Gu, Incheon, 21556 Republic of Korea; 6 Division of Infectious Disease Response, Gyeongnam Regional Center for Disease Control and Prevention, Korea Disease Control and Prevention Agency, 1090, Jungang-daero, Yeonje-gu, Busan, 49596 Republic of Korea; 7 Division of Vectors and Parasitic Disease, Korea Disease Control and Prevention Agency, 187, Osongsaengmyeong 2-ro, Osong-eup, Heungdeok-gu, Cheongju, 28159 Republic of Korea

**Keywords:** *Plasmodium vivax*, Malaria, Republic of Korea, Children, Adolescents

## Abstract

**Background:**

Although *Plasmodium vivax* (*P. vivax)* malaria is in the pre-elimination phase in the Republic of Korea (ROK), it continues to affect children and adolescents, who account for approximately 4–6% of the 300 to 500 annual cases. Despite this, research focusing on *P. vivax* malaria in this particular population remains limited. This study investigates the clinical characteristics of pediatric *P. vivax* malaria in the ROK from 2000 to 2016.

**Methods:**

We retrospectively analyzed pediatric patients aged 0–18 years, diagnosed with *P. vivax* malaria in five hospitals in Goyang City and Seoul. Data on demographics, clinical presentations, treatment regimens, and outcomes were collected. Statistical analyses were performed for comparisons between severe and non-severe cases, across age groups, and assessing trends over time.

**Results:**

A total of 156 pediatric cases of indigenous *P. vivax* malaria were diagnosed. The median patient age was 13 years (men: 64.7%). Severe malaria occurred in 13.5% patients, predominantly in adolescents aged 15–18 years. The most common severe manifestations were jaundice (57.1%) and anemia (33.3%). In the ROK, the treatment regimen for pediatric *P. vivax* malaria involves oral administration of chloroquine at a dose of 25 mg base/kg divided over 3 days, followed by primaquine at a dose of 0.3 mg/kg for 14 days. Although all patients received chloroquine, a higher proportion of younger patients received a dose less than 25 mg/kg (87.5%, 85.5%, and 58.6% of those aged 0–4, 5–14, and 15–18 years, respectively; *p* < 0.001). Parasite clearance time (PCT) increased over the years, suggesting a potential decline in the chloroquine sensitivity of *P. vivax*. No deaths or significant long-term complications were reported.

**Conclusions:**

Pediatric *P. vivax* malaria showed a low incidence of severe cases and no mortality in the ROK. Underdosing of antimalarial drugs was observed, underscoring the need for educating healthcare providers to ensure appropriate dosing. Increasing PCT highlights the need for ongoing surveillance of drug efficacy in this population. Further research on the evolving sensitivity of *P. vivax* and improved treatment protocols is thus essential.

**Supplementary Information:**

The online version contains supplementary material available at 10.1186/s12879-025-10501-9.

## Background

Malaria remains a significant global public health concern and affects a substantial number of individuals annually, particularly in Africa and Asia, where the prevalence of the disease is high. According to the World Health Organization (WHO), approximately 249 million malaria cases were reported worldwide in 2022, with 608,000 deaths [1]. The majority of malaria cases globally are attributed to *Plasmodium falciparum*, which is prevalent in tropical and subtropical areas and is associated with high complication and mortality rates. *Plasmodium vivax (P. vivax)* malaria is the second most prevalent type and is widely distributed in tropical and subtropical regions. Although clinically less severe, it occurs globally in the broadest geographic areas and poses a serious threat to human health and livelihoods.

**Fig. 1:**
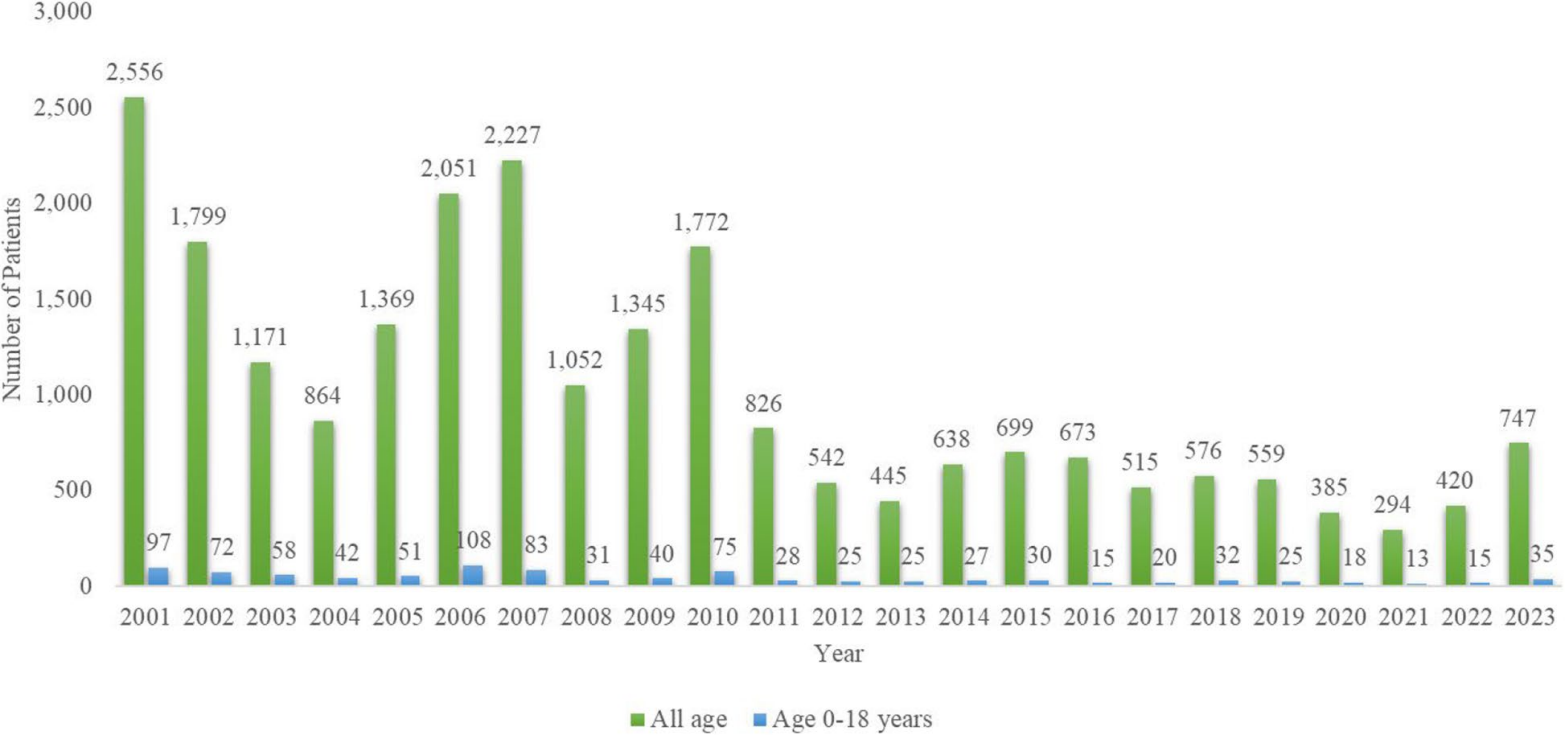
Trends in the number of patients diagnosed with malaria in South Korea by year from 2001 to 2023

The Republic of Korea (ROK) has historically faced endemic malaria, which was associated with significant health challenges until the mid-twentieth century [2]. The effective implementation of malaria prevention and control policies since the 1960s has resulted in a notable reduction in malaria incidence, leading to the ROK being considered a malaria-free country in 1979. However, after the resurgence of vivax malaria near the demilitarized zone (DMZ) in 1993, there was a significant increase in cases, reaching 4,142 in 2000, followed by a decline to 385 in 2013 [3, 4]. Subsequently, there has been a declining trend in recent years, with an annual incidence of around 300 to 500 cases, as reported by the Korea Disease Control and Prevention Agency (KDCA). Among these cases, pediatric patients (i.e., children and adolescents) account for approximately 4–6% of cases annually (Fig. 1). During the 5 years from 2018 to 2022, a total of 125 patients aged 0–19 years were diagnosed with malaria in the ROK, accounting for 6.26% of the total 1998 cases [5, 6].

Among febrile patients suspected or diagnosed with malaria, the majority are referred to referral hospitals for further management. Once diagnosed with *P. vivax* malaria, most adult and pediatric patients receive short-term inpatient treatment and are discharged after achieving defervescence. During the hospitalization period, chloroquine therapy is typically completed, while primaquine is prescribed for outpatient administration to complete the full course. Supportive care, including the administration of antipyretics, blood transfusion, and intravenous fluid therapy, is often provided during inpatient stay.

The guidelines issued by the KDCA recommend oral administration of chloroquine at a dose of 25 mg base/kg divided over 3 days, followed by primaquine at a dose of 0.3 mg/kg for 14 days. However, varying regimens of chloroquine and primaquine have been utilized in clinical practice. Notably, significant variations in primaquine dosing have been observed.

While extensive data exist regarding pediatric *P. vivax* malaria in tropical regions [7–14], there is a lack of information on pediatric *P. vivax* malaria in temperate areas. In the ROK, pediatric malaria cases are geographically restricted and account for a small proportion of total cases, leading to limited clinical data on pediatric *P. vivax* malaria in the ROK. Consequently, many physicians, particularly pediatricians, may lack experience in managing pediatric malaria, despite the disease's continued endemicity.

This study aimed to investigate the clinical characteristics of *P. vivax* malaria in children and adolescents in the ROK from 2000 to 2016 when the malaria incidence was high. The study period was limited to 2016 due to the impact of the COVID-19 pandemic. During the pandemic, the number of malaria cases decreased significantly, epidemiological investigations were incomplete, and hospitalization for patients with malaria became increasingly difficult.

## Methods

### Study sites

**Fig. 2:**
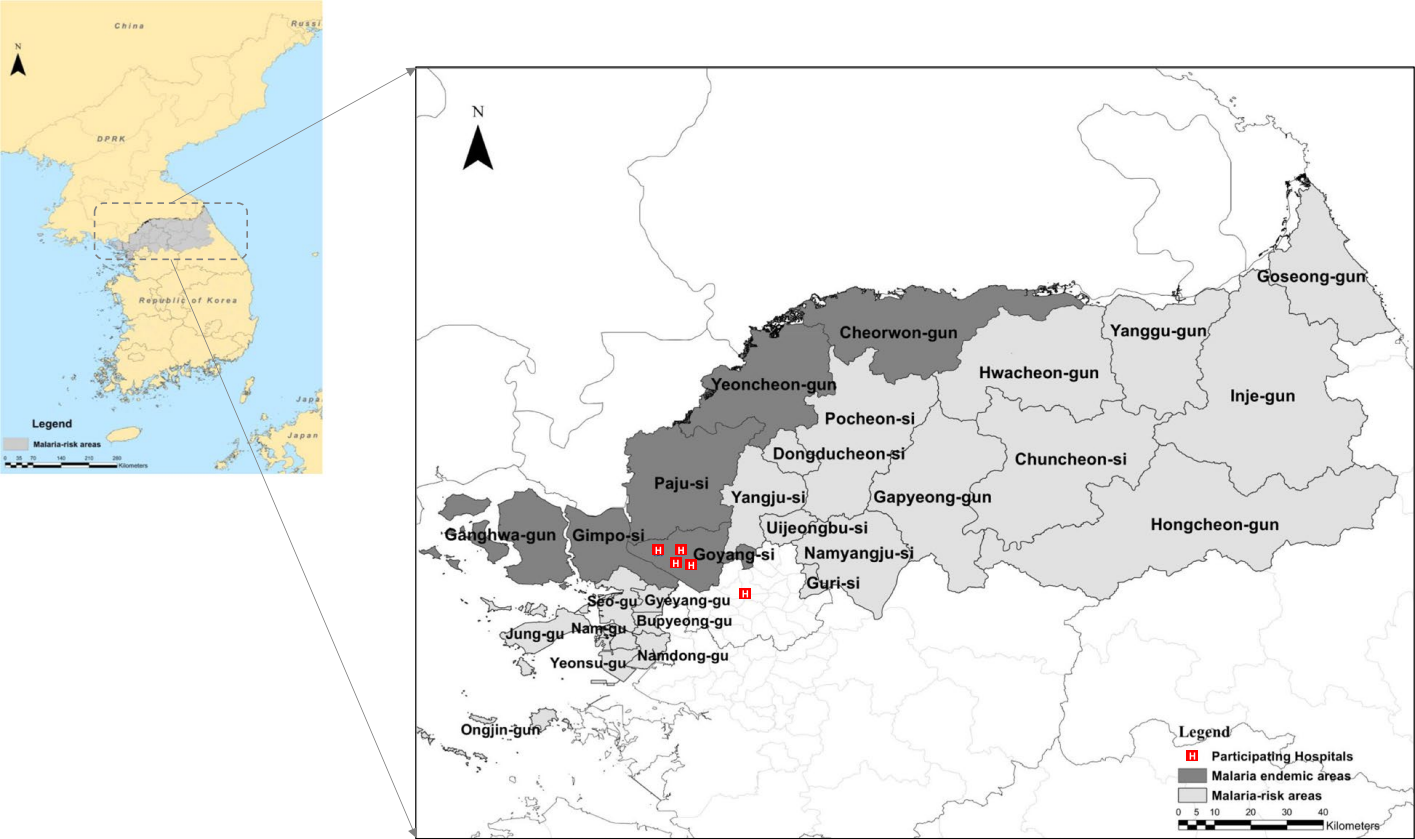
Map showing *Plasmodium vivax* malaria risk areas and participating hospital locations in the Republic of Korea

This retrospective study was conducted in four referral hospitals in Goyang city, Gyeonggi Province, and one tertiary hospital in Seoul. Goyang city is located in the northwestern region of the ROK, close to the DMZ, where malaria is endemic (Fig. 2). The hospitals, with capacities of 600–800 beds, were staffed by infectious disease specialists and pediatricians, and operated laboratories accredited under a national accreditation program. Malaria laboratory investigations were quality-controlled and accredited by the KDCA. Pediatric patients were either referred to the hospital from primary care clinics or presented directly to the hospital's emergency department, and the decision regarding hospitalization was made at the discretion of the pediatricians.

### Study patients and data collection

The study population consisted of patients under 19 years of age who were diagnosed with indigenous *P. vivax* malaria via peripheral blood smear between 2000 and 2016. Patients with imported malaria cases were excluded from the analysis. Considering that *P. vivax* malaria is the only endemic malaria species in the ROK, all cases caused by non-vivax *Plasmodium* species were classified as imported malaria. In the ROK, all malaria cases are legally required to be reported, and an epidemiological investigation is conducted for each reported case. Cases with epidemiological links to malaria-endemic regions were categorized as imported malaria.

Data were collected by the research team using a standardized case report form. Medical records were reviewed to investigate the following variables: sex, age, weight, underlying conditions, history of previous malaria infection, date of malaria diagnosis, admission date, discharge date, intensive care unit admission status, initial vital signs, symptoms, laboratory test results, malaria treatment regimens and dosages, time of chloroquine administration, time of confirmed *P. vivax* clearance from the peripheral blood smear, fever status, malaria relapse, and mortality. The status of glucose-6-phosphate dehydrogenase (G6PD) deficiency was not included in the data collection for this study, as G6PD deficiency is rare in Koreans [15, 16].

### Definitions

Severe *P. vivax* malaria was defined using the same criteria as those used for falciparum malaria, except for parasite density thresholds, following the WHO guidelines [17]. Namely, the WHO criteria included: impaired consciousness (Glasgow Coma Score < 11 or Blantyre coma score < 3), metabolic acidosis (base deficit of > 8 mEq/L, or a plasma bicarbonate of < 15 mmol/L, or venous plasma lactate > 5 mmol/L), hypoglycemia (blood or plasma glucose < 40 mg/dL), severe malarial anemia (hemoglobin < 5 g/dL or a hematocrit of < 15% in children < 12 years of age, < 7 g/dL and < 20%, respectively, in adults), renal impairment (plasma or serum creatinine > 3 mg/dL or blood urea > 60 mg/dL), jaundice (plasma or serum total bilirubin > 3 mg/dL), pulmonary edema (radiologically confirmed, or oxygen saturation < 92% on room air with a respiratory rate > 30/min, often with chest indrawing and crepitations on auscultation), significant bleeding (including recurrent or prolonged bleeding from nose, gums or venipuncture sites; hematemesis or melena), and compensated shock (defined as capillary refill ≥ 3 s, or temperature gradient on leg, but no hypotension), or decompensated shock (defined as systolic blood pressure < 70 mmHg in children or < 80 mmHg in adults with evidence of impaired perfusion). Fever was defined as a body temperature greater than 37.8 °C. Severe thrombocytopenia was defined as a platelet count < 50,000/μL. Hepatic dysfunction was defined as an aspartate aminotransferase or alanine aminotransferase value exceeding three times the normal upper limit or total bilirubin concentration exceeding 3 mg/dL. The parasite clearance time (PCT) was defined as the time in hours from chloroquine administration to the first negative blood smear for parasites, after which all subsequent follow-up smears were negative, and was determined from daily malaria smears obtained during treatment. *P. vivax* relapse was defined as reappearance of *P. vivax* parasitemia more than 30 days after starting primaquine.

### Statistical analysis

Statistical analysis was conducted using the R 4.3.0 software (R Foundation for Statistical Computing, Vienna, Austria). The Mann–Whitney U test was employed to compare continuous variables between the severe malaria and non-severe malaria groups, while the Pearson χ^2^ test or Fisher's exact test was used to compare categorical variables. For comparing between age groups or time of malaria diagnosis, the Jonckheere–Terpstra test was used for continuous variables, while the Cochran–Armitage Trend test was used for categorical variables. The Kruskal–Wallis H test was used to analyze the age distribution across the different time periods. A *p*-value of less than 0.05 was regarded as statistically significant.

Although this was a retrospective study and no prospective sample size calculation was performed, a post-hoc power analysis was conducted to assess whether our sample size of 156 patients was sufficient for detecting significant differences in key outcomes. Based on an estimated effect size of 0.3 for differences in severe malaria incidence between age groups, a significance level of 0.05, and a power of 80%, we calculated that a minimum sample size of 111 patients would be required. Therefore, our study's sample size was deemed adequate for inferential statistical analyses.

## Results

### Baseline characteristics

During the study period from 2000 to 2016, a total of 156 cases of indigenous *P. vivax* malaria were diagnosed in children and adolescents. The median age was 13 years (interquartile range [IQR], 8–16 years), and 101 (64.7%) patients were men. A total of 151 (96.8%) patients were hospitalized. Fever was present in 138 (88.5%) patients and severe thrombocytopenia (platelet count ≤ 50,000/μL) in 35.3% of patients. Hepatic dysfunction occurred in 14.9% of patients (Table 1).

**Table 1:** Comparison of severe versus non-severe *Plasmodium vivax* malaria characteristics

Variables	Total (*N* = 156)	Non-severe malaria (*N* = 135)	Severe malaria (*N* = 21)	*p*-value
Age, years, median [IQR]	13 [8–16]	12 [7.5–16]	15 [13–17]	0.024^c^
Male sex, n (%)	101 (64.7)	91 (67.4)	10 (47.6)	0.128^d^
Body weight, kg, median [IQR]	50.0 [28.1–61.5]	47.0 [25.0–61.0]	58.4 [49.5–67.2]	0.037^c^
Comorbidity, n (%)	14 (9.0)	11 (8.1)	3 (14.3)	0.406^d^
**Study sites, n(%)**				0.215^d^
Site 1	13 (8.3)	9 (6.7)	4 (19.0)	
Site 2	11 (7.1)	11 (8.1)	0 (0)	
Site 3	76 (48.7)	65 (48.1)	11 (52.4)	
Site 4	47 (30.1)	41 (30.4)	6 (28.6)	
Site 5	9 (5.8)	9 (6.7)	0 (0)	
Admission, n (%)	151 (96.8)	130 (96.3)	21 (100.0)	1.000^d^
ICU admission, n (%)	3 (1.9)	0 (0.0)	3 (14.3)	0.002^d^
**Clinical features, n (%)**				
Fever	138 (88.5)	117 (86.7)	21 (100.0)	0.134^d^
Shock ^a^	3 (2.0)	0 (0.0)	3 (14.3)	N/A
Jaundice ^a^	12 (7.7)	0 (0.0)	12 (57.1)	N/A
Severe anemia ^a^	7 (4.5)	0 (0.0)	7 (33.3)	N/A
Severe thrombocytopenia ^b^	55 (35.3)	44 (32.6)	11 (52.4)	0.128^d^
Hepatic dysfunction ^e^	23 (14.9)	11 (8.3)	12 (57.1)	< 0.001^d^
Hyperbilirubinemia-only ^f^	4 (2.6)	0 (0)	4 (19.0)	< 0.001^d^
**Laboratory data (worst), median [IQR]**				
Hemoglobin, g/dL	9.9 [8.8–11.4]	10.3 [9.0–11.5]	8.3 [6.3–9.5]	< 0.001^c^
Platelet count, 10^3^/μL	61 [43–94.5]	62.5 [45–96]	43 [31–74]	0.009^c^
Creatinine, mg/dL	0.8 [0.7–1.0]	0.8 [0.6–1.0]	1.0 [0.8–1.2]	0.028^c^
AST, IU/L	38 [28–55]	36 [28–52]	45 [36–140]	0.024^c^
ALT, IU/L	35 [23–71]	35 [22–66]	40 [31–77]	0.117^c^
Total bilirubin, mg/dL	1.4 [1.1–2.0]	1.2 [1.1–1.9]	3.1 [2.2–3.4]	< 0.001^c^
Glucose, mg/dL	108 [97–120]	108 [97–121]	102 [92–115]	0.241^c^

*IQR* interquartile range, *ICU* intensive care unit, *IU* international unit, *AST* aspartate aminotransferase, *ALT* alanine aminotransferase

^a^These variables were not included in the analysis because they are part of the definition of severe malaria

^b^Severe thrombocytopenia was defined as a platelet count < 50,000/μL

^c^The *p*-value was calculated using the Mann–Whitney U test

^d^The *p*-value was calculated using the Pearson χ^2^ test or Fisher's exact test

^e^Hepatic dysfunction category includes cases of hyperbilirubinemia only

^f^Refers to cases where total bilirubin levels were elevated to ≥ 3 mg/dL without accompanying AST or ALT elevation

### Comparison of patients with severe and non-severe *P. vivax* malaria

Among the patients diagnosed with *P. vivax* malaria, 21 (13.5%) met the criteria for severe *P. vivax* malaria. An additional figure shows this in more detail (see Additional file 1). The median age of the patients with severe malaria was significantly higher than that of patients with non-severe malaria (15 years [IQR 13–17] vs. 12 years [IQR 7.5–16], *p* = 0.024). Only one patient (4.8%) in the severe malaria group was aged ≤ 4 years, whereas 61.1% (13/21) patients were aged between 15 and 18 years. A comparison of the clinical features between the severe and non-severe cases is presented in Table 1. The clinical manifestations of severe malaria included shock, jaundice, and anemia in 14.3%, 57.1%, and 33.3% of patients, respectively (Table 1). No cases of impaired consciousness, metabolic acidosis, hypoglycemia, renal impairment, pulmonary edema, or significant bleeding were observed. None of the cases had a history of previous malaria infection.

### Comparison of clinical characteristics by age group

Patients were categorized into three age groups: 0–4 years, 5–14 years, and 15–18 years. A statistically significant trend was observed in the prevalence of severe malaria, with the highest incidence in the oldest age group (6.2% vs. 9.1% vs. 20.6%, *p* = 0.039). Additionally, the incidence of jaundice was notably higher in the older age group (0.0% vs. 3.9% vs. 14.3%, *p* = 0.012). All patients received chloroquine; however, a significant proportion of younger patients received doses below 25 mg/kg (87.5% vs. 85.5% vs. 58.6%, *p* < 0.001). However, more than half of the patients in the oldest age group also received a chloroquine dose below the recommended level. Most patients were treated with primaquine, with only two exceptions. In the 15–18 years age group, 44.8% of patients received a primaquine dose below the recommended 0.25 mg/kg. PCT was significantly prolonged in the 15–18 years age group (110.5 h [IQR 77.5–184.5] vs. 81.5 h [IQR 60–118] vs. 78 h [IQR 60–116], *p* = 0.008), with a higher proportion of cases demonstrating confirmed parasitemia 96 h post-treatment initiation (64.9% vs. 41.9% vs. 37.5%, *p* = 0.026). There were no significant differences in relapse rates among the groups, and no fatalities were reported (Table 2).

**Table 2:** Comparison of clinical characteristics, treatment, and treatment responses by age group

Variables	Age 0–4 years (*N* = 16)	Age 5–14 years (*N* = 77)	Age 15–18 years (*N* = 63)	*p*-value
Male sex, n (%)	8 (50.0)	29 (37.7)	18 (28.6)	0.088^d^
Body weight, kg, median [IQR]^a^	13.4 [11.2–15.0]	35.8 [25.0–50.0]	63.5 [55.7–70.0]	< 0.001^c^
Comorbidity, n (%)	3 (18.8)	6 (7.8)	5 (7.9)	0.335^d^
Admission, n (%)	16 (100.0)	75 (97.4)	60 (95.2)	0.292^d^
ICU admission, n (%)	1 (6.2)	2 (2.6)	0 (0.0)	0.085^d^
**Clinical features, n (%)**				
Fever	14 (87.5)	67 (87.0)	57 (90.5)	0.580^d^
Severe malaria	1 (6.2)	7 (9.1)	13 (20.6)	0.039^d^
Shock	1 (7.1)	1 (1.3)	1 (1.6)	0.374^d^
Jaundice	0 (0.0)	3 (3.9)	9 (14.3)	0.012^d^
Severe anemia	1 (6.2)	3 (3.9)	3 (4.8)	0.948^d^
Severe thrombocytopenia	3 (18.8)	23 (29.9)	29 (46.0)	0.014^d^
Hepatic dysfunction ^e^	1 (6.2)	7 (9.2)	15 (24.2)	0.013^d^
Hyperbilirubinemia-only ^f^	0 (0)	2 (2.6)	2 (3.2)	1.000^d^
**Treatment**				
Chloroquine (CQ), n (%)	16 (100.0)	77 (100.0)	63 (100.0)	1.000^d^
CQ, mg base/kg, median [IQR]^a^	19.6 [18.7–24.0]	20.4 [18.8–24.3]	23.1 [21.4–26.8]	< 0.001^c^
CQ < 25 mg base/kg, n (%) ^a^	14/16 (87.5)	65/76 (85.5)	34/58 (58.6)	< 0.001^d^
Primaquine, n (%)	16 (100.0)	75 (97.4)	63 (100.0)	0.506^d^
Primaquine, mg base/kg, median [IQR**]**^a^	0.3 [0.3–0.5]	0.3 [0.3–0.5]	0.2 [0.2–0.3]	< 0.001^c^
Primaquine < 0.25 mg base/kg, n (%) ^a^	1/16 (6.2)	14/76 (18.4)	26/58 (44.8)	< 0.001^d^
**Treatment response**				
PCT^b^, hours, median [IQR]	78 [60–116]	81.5 [60–118]	110.5 [77.5–184.5]	0.008^c^
PCT^b^ > 72 h, n (%)	9/16 (56.2)	38/62 (61.3)	29/37 (78.4)	0.065^d^
PCT^b^ > 96 h, n (%)	6/16 (37.5)	26/62 (41.9)	24/37 (64.9)	0.026^d^
Relapse, n (%)	2 (12.5)	1 (1.3)	1 (1.6)	0.083^d^
Death, n (%)	0 (0.0)	0 (0.0)	0 (0.0)	1.000^d^

*IQR* interquartile range, *ICU* intensive care unit, *PCT* parasite clearance time

^a^Includes data from 150 patients with body weight results

^b^Includes data from 115 patients with PCT results

^c^The *p*-value was calculated using the Jonckheere–Terpstra test

^d^The *p*-value was calculated using the Cochran–Armitage Trend test

^e^Hepatic dysfunction category includes cases of hyperbilirubinemia only

^f^Refers to cases where total bilirubin levels were elevated to ≥ 3 mg/dL without accompanying AST or ALT elevation

### PCT trend by the time of malaria diagnosis

When the patients were divided into three groups based on the period of *P. vivax* malaria diagnosis (2000–2005, 2006–2010, and 2011–2016), the median weight of the patients was significantly higher in the more recent periods (45.0 kg [IQR 20.5–61.0] vs. 47.0 kg [IQR 28.1–58.0] vs. 59.2 kg [IQR 44.0–68.7], *p* = 0.007). However, the age distribution did not show a statistically significant difference (*p* = 0.163). No significant differences were observed across these periods with regard to malaria treatment. However, PCT levels appeared to increase over time (73 h [IQR 56–103] vs. 102 h [IQR 64–133] vs. 110 h [IQR 81–207], *p* = 0.001). Similarly, the proportion of patients in whom parasitemia persisted 72 and 96 h after the initiation of antimalarial treatment showed a rising trend. Conversely, the relapse rate gradually declined, although this difference was not statistically significant (Table 3). The administration of chloroquine at a dose exceeding 25 mg base/kg was not associated with PCT or parasite clearance within 72 h of initiating treatment (Table 4).

**Table 3:** Comparison of malaria treatment and treatment responses by time of malaria diagnosis

Variables	2000–2005 (*N* = 51)	2006–2010 (*N* = 67)	2011–2016 (*N* = 38)	*p*-value
Age, y, median [IQR]	13 [6–16]	12 [8–16]	13 [11–16]	0.136^c^
Age, n (%)				0.163 ^e^
0–4 years	9 (17.6)	6 (9.0)	1 (2.6)	
5–14 years	23 (45.1)	31 (46.3)	23 (60.5)	
15–18 years	19 (37.3)	30 (44.8)	14 (36.8)	
Male sex, n (%)	32 (62.7)	45 (67.2)	24 (63.2)	0.926^d^
Body weight, kg, median [IQR]^a^	45.0 [20.5–61.0]	47.0 [28.1–58.0]	59.2 [44.0–68.7]	0.007^c^
Age 0–4 years	12.0 [11.0–14.0]	13.9 [11.8–15.5]	18.1 [18.1–18.1]	0.054^c^
Age 5–14 years	35.0 [25.0–50.0]	30.2 [24.0–44.0]	48.0 [29.5–60.8]	0.053^c^
Age 15–18 years	65.0 [60.0–67.4]	58.0 [53.1–68.2]	68.7 [61.0–73.5]	0.170^c^
**Treatment**				
Chloroquine (CQ), n (%)	51 (100.0)	67 (100.0)	38 (100.0)	1.000^d^
CQ, mg base/kg, median [IQR]^a^	20.3 [18.8–23.1]	23.9 [18.9–25.6]	22.1 [20.3–24.6]	0.050^c^
CQ < 25 mg base/kg, n (%) ^a^	40/48 (83.3)	44/65 (67.7)	29/37 (78.4)	0.493^d^
Primaquine, n (%)	51 (100.0)	66 (98.5)	37 (97.4)	0.269^d^
Primaquine, mg base/kg, median [IQR] ^a^	0.3 [0.2–0.3]	0.3 [0.3–0.4]	0.3 [0.2–0.5]	0.952^c^
Primaquine < 0.25 mg base/kg, n (%) ^a^	15/48 (31.2)	13/65 (20.0)	13/37 (35.1)	0.806^d^
**Treatment response**				
PCT^b^, hours, median [IQR]	73 [56–103]	102 [64–133]	110 [81–207]	0.001^c^
PCT^b^ > 72 h, n (%)	15/31 (48.4)	36/52 (69.2)	25/32 (78.1)	0.013^d^
PCT^b^ > 96 h, n (%)	8/31 (25.8)	28/52 (53.8)	20/32 (62.5)	0.004^d^
Relapse, n (%)	3 (5.9)	1 (1.5)	0 (0.0)	0.072^d^
Death, n (%)	0 (0.0)	0 (0.0)	0 (0.0)	1.000^d^

*IQR* interquartile range, *ICU* intensive care unit, *PCT* parasite clearance time

^a^Includes data from 150 patients with body weight results

^b^Includes data from 115 patients with PCT results

^c^The *p*-value was calculated using the Jonckheere–Terpstra test

^d^The *p*-value was calculated using the Cochran–Armitage Trend test

^e^The *p*-value was calculated using the Kruskal–Wallis H test

**Table 4:** Analysis of the relationship between chloroquine dosing and parasitemia clearance times

Variables	CQ ≥ 25 mg base/kg (*N* = 25)	CQ < 25 mg base/kg (*N* = 87)	*p*-value
PCT, hours, median [IQR]	92.0 [64.0117.5]	89.0 [64.0;126.0]	0.928^a^
PCT > 72 h, n (%)	17 (68.0)	57 (65.5)	1.000^b^
PCT > 96 h, n (%)	12 (48.0)	42 (48.3)	1.000^b^

*CQ* Chloroquine, *IQR* interquartile range, *PCT* parasite clearance time

^a^The *p*-value was calculated using the Mann–Whitney U test

^b^The *p*-value was calculated using the Pearson χ^2^ test or Fisher's exact test

## Discussion

*P. vivax* malaria in the ROK is currently in the pre-elimination phase, with approximately 300–500 cases reported annually. Of these, only approximately 6% are pediatric infections [5]. Although the overall number of pediatric cases is relatively low, severe manifestations may occur in children. However, research focusing specifically on *P. vivax* malaria in pediatric populations remains limited. In particular, data on the temperate strains of *P. vivax*, which can cause malaria seasonally in some temperate regions, including developed countries such as the ROK, are lacking. Therefore, our study provides a comprehensive analysis of these data and offers valuable insights into this disease in the pediatric population.

*P. vivax* malaria is known to cause debilitating febrile illness with progressive anemia and, in some cases, severe disease similar to that caused by *P. falciparum* malaria [14]. The risk of severe disease and case fatality rates among pediatric patients are inconsistent and remain undefined [18]. While findings vary across studies, one study from Colombia reported that 23.8% of patients with *P. vivax* malaria under the age of 15 years developed severe malaria, and the case fatality rate of *P. vivax* monoinfection was 0.01% [19]. Another study from India on children hospitalized with malaria found that the disease severity was higher in *P. vivax* infections (63.1%) than in *P. falciparum* (42.7%) and mixed infections (40%). Additionally, the case-fatality rate among children with *P. vivax* monoinfection was 3.9% (4/103) [13].

In contrast, only 13.5% of pediatric patients with *P. vivax* malaria had severe malaria in our study, with no fatalities being reported. Moreover, an age-based analysis revealed that only one case of severe malaria was diagnosed in a child under 5 years of age, with the proportion of severe cases increasing significantly with age. This contrasts with the findings of Genton et al., who reported the highest rates of severe *P. vivax* malaria in children aged 0–2 years, with the rates decreasing with increasing age [20].

The low incidence of severe malaria and the absence of fatalities may be attributed to differences in healthcare accessibility. In the ROK, even non-severe cases of malaria are typically hospitalized for short-term treatment following diagnosis. Consequently, many non-severe cases were included in our study. In contrast, studies from other countries often included only hospitalized patients presenting with severe manifestations. It is also possible that differences between temperate and tropical strains of *P. vivax* malaria contributed to the observed findings. Reportedly, tropical strains are associated with faster relapse intervals, typically 3–6 weeks, whereas temperate strains exhibit longer relapse intervals of 6–12 months [21, 22]. Additionally, tropical strains tend to present with higher parasitemia and more frequent multiple invasions of erythrocytes, whereas temperate strains show relatively fewer multiple invasions [23]. Since the extent of multiple erythrocyte invasions has been linked to anemia [24], these strain differences may influence the severity or fatality of *P. vivax* malaria. Further research is needed to better elucidate the impact of strain differences on disease outcomes. Finally, in the ROK, G6PD deficiency is rare, allowing nearly all patients to receive primaquine therapy. This reduces the likelihood of relapse and associated complications, such as anemia, that can result from repeated relapses.

In children with severe malaria, jaundice was the most common manifestation, followed by anemia and shock, a pattern that differs from that in other countries where severe anemia is more frequently observed [18]. Additionally, Bhattacharjee et al. reported respiratory findings in 13% of pediatric patients with *P. vivax* malaria in a cohort of 168 children [10]. However, we did not observe any patients with respiratory findings, which is another significant difference.

In our previous study on *P. vivax* malaria in adults in the ROK, 18.7% (255 of 1,366) of patients with *P. vivax* malaria were classified as having severe malaria. This proportion is higher than that observed in pediatric patients with *P. vivax* malaria, indicating that the severity is lower in children and tends to increase with age. Among adults with severe *P. vivax* malaria, shock was the most common complication, accounting for 45.6% of the cases (115/252) [25]. In contrast, jaundice was the most frequent complication in pediatric patients. While the number of patients with pulmonary manifestations was low in both adults and children, these findings highlight the distinct clinical characteristics of pediatric malaria in the ROK compared with those in adults.

In the ROK, the standard treatments for *P. vivax* malaria are chloroquine and primaquine. Although there is a case report of chloroquine resistance [26], chloroquine remains an effective treatment option. Consequently, all patients in our study were treated with chloroquine, and all patients except for two received primaquine as part of their radical cure. However, a significant proportion of patients were administered chloroquine at doses lower than the recommended amount, and younger patients were more likely to receive suboptimal doses. In contrast, underdosing of primaquine was more common in older patients.

As highlighted in a study by Commons et al., there has been an ongoing debate regarding the dosing of chloroquine in pediatric patients and the associated treatment responses [27]. In our study, we evaluated the administered doses of chloroquine and their effects by assessing the PCT and proportion of patients with persistent parasitemia at 72 h post-treatment. Nevertheless, no statistically significant correlation was identified between chloroquine dosing and either the PCT or rate of parasitemia at 72 h post-treatment. It is noteworthy that despite a higher proportion of patients aged 15 years and older receiving doses exceeding the WHO-recommended chloroquine dose of 25 mg/kg, their PCT was longer. Conversely, patients under 15 years of age who received relatively lower doses, exhibited shorter PCTs. This observation suggests that factors other than the dose may influence the treatment response in different age groups.

Given that the median body weight was 63.5 kg (IQR 55.7–70.0) in the 15–18 years age group, it is probable that a significant proportion of individuals in this age category received the standard adult dosing regimen for chloroquine. This may account for the relatively higher proportion of patients who received an appropriate dosage, given that their body weights were more closely aligned with the adult dosing guidelines. In contrast, for children under the age of 15 years, dosing must be calculated based on body weight. The KDCA malaria treatment guidelines recommend doses based on chloroquine base; however, the drug actually used in clinical practice is hydroxychloroquine. A 200 mg dose of hydroxychloroquine is equivalent to 155 mg of chloroquine base. It is possible that a lack of awareness regarding the difference in dosage between hydroxychloroquine and chloroquine base may have led to some dosing errors. Despite the trend observed in adults [28] where an increase in chloroquine underdosing correlated with longer PCTs, this pattern was not observed in pediatric patients.

In the present study, the trend of increasing PCT levels over time was consistent with that observed in studies conducted in adults during the same period [28]. It can be inferred that the chloroquine sensitivity of the *Plasmodium* parasite decreases over time; however, laboratory evidence to confirm this hypothesis is still lacking, indicating the need for further research.

This study has a few limitations. First, as a retrospective study, it is subject to inherent limitations, such as missing data on weight and smear follow-up, which led to the exclusion of some cases from the analysis. Additionally, the duration of primaquine administration was not verified. In the ROK, cases of *P. vivax* malaria can relapse more than 1 year after the initial infection. However, as follow-up beyond 1 year was not feasible, the relapse status of the patients included in this study may not have been tracked with complete accuracy. Second, the small sample size, particularly the limited number of patients under 5 years of age, poses challenges for generalizing our study results. A post-hoc power analysis confirmed that the sample size was appropriate for the analysis. However, differences in the number of individuals between groups make it difficult to completely rule out the possibility of a type II error. Third, although clinical aspects were analyzed, the lack of supporting laboratory evidence presents limitations in interpreting the findings. Nevertheless, this study is significant because, to the best of our knowledge, it is the first to analyze the clinical characteristics, treatment trends, and outcomes of pediatric patients with *P. vivax* malaria in the ROK.

## Conclusions

Pediatric *P. vivax* malaria in the ROK demonstrated an increase in the proportion of severe cases with age, although no complications or mortality were observed. Underdosing of antimalarial medications was noted frequently in children, highlighting the need to educate healthcare providers to ensure appropriate dosing. Furthermore, the potential decline in the chloroquine sensitivity of Korean *P. vivax* warrants further investigation.

## Supplementary Information

Additional file 1: Supplementary Figure 1. Number of uncomplicated versus severe cases by different years.

## Data Availability

The datasets used and/or analysed during the current study are available from the corresponding author on reasonable request.

## Abbreviations

DMZ: Demilitarized zone
IQR: Interquartile range
KDCA: Korea Disease Control and Prevention Agency
PCT: Parasite clearance time
ROK: Republic of Korea
WHO: World Health Organization
