# Supplementary figures and images for: Clinical characteristics of Plasmodium vivax malaria infection in children and adolescents in the Republic of Korea during the period 2000 to 2016: a retrospective study
Source: BMC Infect Dis. 2025 Jan 21;25:93. doi: 10.1186/s12879-025-10501-9 (PMC11748960; doi:10.1186/s12879-025-10501-9)

**Supplementary Figure 1. Number of uncomplicated versus severe cases by different years.**

**
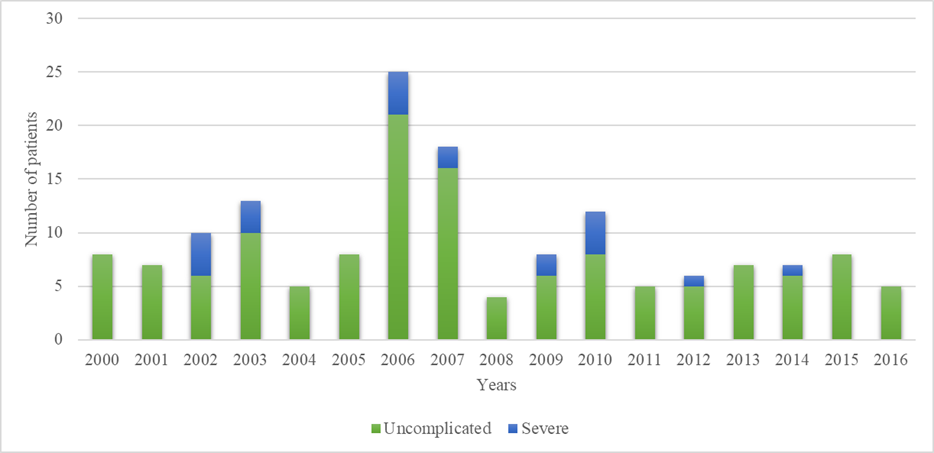
**

Supplement: Supplementary file 1 — Additional file 1: Supplementary Figure 1. Number of uncomplicated versus severe cases by different years. [file 12879_2025_10501_MOESM1_ESM.docx]
